# Clinical Method Applied to Focused Ultrasound: The Case of Wells’ Score and Echocardiography in the Emergency Department: A Systematic Review and a Meta-Analysis

**DOI:** 10.3390/medicina57080766

**Published:** 2021-07-28

**Authors:** Lorenzo Falsetti, Vincenzo Zaccone, Alberto M. Marra, Nicola Tarquinio, Giovanna Viticchi, Mattia Sampaolesi, Francesca Riccomi, Laura Giovenali, Consuelo Ferrini, Gianluca Moroncini, Cinzia Nitti, Aldo Salvi

**Affiliations:** 1 Internal and Subintensive Medicine Department, Azienda Ospedaliero-Universitaria “Ospedali Riuniti” di Ancona, 60100 Ancona, Italy; vincenzozaccone@libero.it (V.Z.); cinzia_nitti@libero.it (C.N.); aldo.salvi25@gmail.com (A.S.); 2 Department of Translational Medical Sciences, “Federico II” University, 80100 Naples, Italy; alberto_marra@hotmail.it; 3 Internal Medicine Department, INRCA-IRCSS Ancona, 60027 Osimo (Ancona), Italy; n.tarquinio@inrca.it; 4 Clinical and Experimental Medicine Department, Neurological Clinic, Azienda Ospedaliero-Universitaria “Ospedali Riuniti” di Ancona, 60100 Ancona, Italy; viticchi.g@gmail.com; 5 Emergency Medicine Residency Program, Marche Polytechnic University, 60100 Ancona, Italy; sampaolesimattia@gmail.com (M.S.); riccomi.francesca@gmail.com (F.R.); g.laura9994@gmail.com (L.G.); consueloferrini@hotmail.com (C.F.); 6 Clinical and Experimental Medicine Department, Clinica Medica, Azienda Ospedaliero-Universitaria “Ospedali Riuniti” di Ancona, 60100 Ancona, Italy; g.moroncini@univpm.it

**Keywords:** pulmonary embolism, echocardiography, diagnosis, critical care, emergency department

## Abstract

*Background and Objectives:* bedside cardiac ultrasound is a widely adopted method in Emergency Departments (ED) for extending physical examination and refining clinical diagnosis. However, in the setting of hemodynamically-stable pulmonary embolism, the diagnostic role of echocardiography is still the subject of debate. In light of its high specificity and low sensitivity, some authors suggest that echocardiographic signs of right ventricle overload could be used to rule-in pulmonary embolism. In this study, we aimed to clarify the diagnostic role of echocardiographic signs of right ventricle overload in the setting of hemodynamically-stable pulmonary embolism in the ED. *Materials and Methods:* we performed a systematic review of literature in PubMed, Web of Science and Cochrane databases, considering the echocardiographic signs for the diagnosis of pulmonary embolism in the ED. Studies considering unstable or shocked patients were excluded. Papers enrolling hemodynamically stable subjects were selected. We performed a diagnostic test accuracy meta-analysis for each sign, and then performed a critical evaluation according to pretest probability, assessed with Wells’ score for pulmonary embolism. *Results:* 10 studies were finally included. We observed a good specificity and a low sensitivity of each echocardiographic sign of right ventricle overload. However, once stratified by the Wells’ score, the post-test probability only increased among high-risk patients. *Conclusions:* signs of echocardiographic right ventricle overload should not be used to modify the clinical behavior in low- and intermediate- risk patients according to Wells’ score classification. Among high-risk patients, however, echocardiographic signs could help a physician in detecting patients with the highest probability of pulmonary embolism, necessitating a confirmation by computed tomography with pulmonary angiography. However, a focused cardiac and thoracic ultrasound investigation is useful for the differential diagnosis of dyspnea and chest pain in the ED.

## 1. Introduction

Integrated cardiac and lung ultrasounds, performed at the bedside by the emergency medicine physicians, are becoming the standard method to improve diagnoses among critically ill patients admitted to the emergency department (ED) [1], especially in patients affected by undifferentiated dyspnea [2,3], shock, chest pain and cardiac arrest [4]. Bedside ultrasound can improve the differential diagnosis of shock and acute heart failure. Furthermore, it has been proposed as a potential means of diagnosing pulmonary embolism (PE), especially among patients with specific contraindications to second-level imaging, as contrast-enhanced computed tomography angiography (CTPA) or V/Q scan. However, while the presence of right ventricle (RV) dilatation and hypokinesis is deemed to be diagnostic for PE in patients admitted with shock, cardiac arrest or persistent hypotension [5], the role of echocardiography as a diagnostic procedure in haemodynamically stable patients admitted to the ED is less clear. Some authors have suggested the potential role of echocardiography in the rule-in of PE, especially in those with a high pre-test probability [6]. With this systematic review and meta-analysis, with subsequent analysis of post-test probability, we aimed to clarify the role of echocardiography in haemodynamically stable subjects admitted to the ED with a potential PE diagnosis.

## 2. Materials and Methods

*Systematic Review of Literature:* we conducted a systematic review of the literature, considering papers published in the English language from 1 January 1990 to 31 December 2020. We performed the literature search in the following repositories: PubMed/MedLine, Web of Sciences and Cochrane Database. The research strategy is described in Supplementary Materials Table S1. The systematic review and its results are compliant to PRISMA 2020 reporting guidelines [7]. Four independent reviewers (L.F., V.Z., L.G. and C.F.) reviewed the selected literature and chose the references adopted for further analyses.

*Inclusion and Exclusion Criteria:* We only selected papers in the English language reporting data in a form that allowed the calculation of sensitivity and specificity, dealing of transthoracic echocardiography as a diagnostic mean for PE in the ED. We excluded works dealing with animals and humans aged less than 18 years old as well as reviews, case reports and small-sampled cohort studies (less than 40 patients). We also excluded all the papers that adopted echocardiography to stratify PE prognosis, that conducted studies outside of an ED (particularly those where the study was conducted in cohorts of inpatients, such as cardiology or intensive care units’ inpatients), that adopted transesophageal echocardiography or dealt with less-available techniques (for example, tissue-doppler imaging). Moreover, we excluded the studies performed in patients with COVID-19, shock, refractory hypotension or thrombus-in-transit. All the retrieved papers were saved into EndNote 20 for MacOS Systems, and duplicate records were removed through this software.

*Quality Analysis, Data Collection and Processing:* two reviewers (G.L. and F.C.) independently assessed the quality of the selected papers, adopting a validated tool (QUADAS-2) [8]. An absence of agreement between the two reviewers was resolved via consensus after a discussion with members of the team (F.L., Z.V., G.V., A.M.M.). The data retrieved from the selected studies were included into the RevMan 5.4 software for MacOS systems. We then calculated HSROC and bivariate model parameters to calculate pooled sensitivity, specificity, positive and negative likelihood ratios, and diagnostic odds ratios with the MetaDTA software [9].

*Wells Score and Post-Test Probability:* According to its original definition [10], Wells’ score for PE can stratify subjects at low-, intermediate- or high-risk considering only clinical variables, as shown in Table 1. According to Wells’ original study, PE prevalence in an ED population is 1.3% among low-risk patients, 16.2% in the intermediate-risk group and 40.6% in the high-risk group [10]. We obtained Fagan’s diagram and calculated the post-test probabilities of each echocardiographic sign according to Wells’ score adopting the diagnostic test calculator (version 2010042101) by Alan Schwartz [11].

## 3. Results

**Figure 1:**
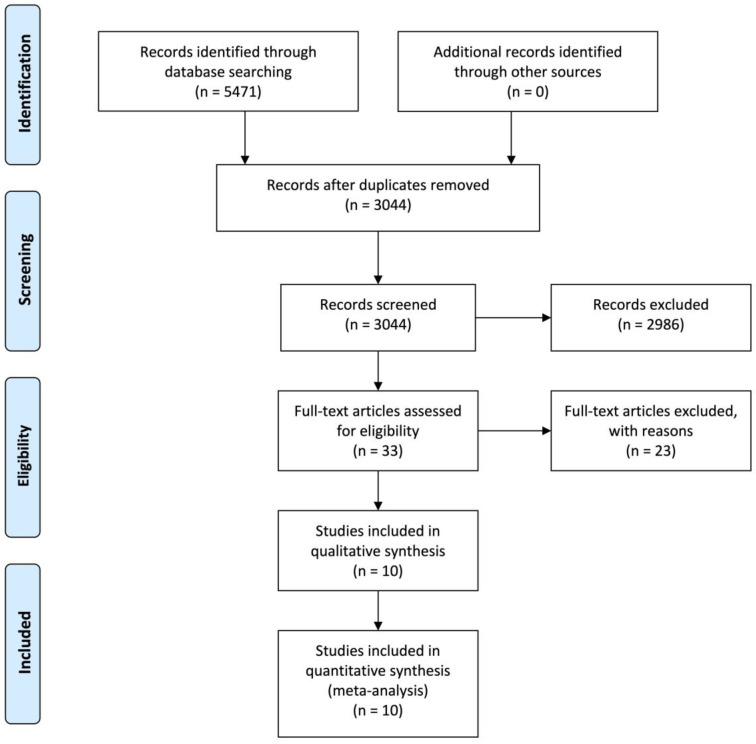
Flow diagram for paper selection.

Our research strategy retrieved 5471 papers, 3044 of which resulted after duplicate removal. We excluded 1198 case reports, 1095 unrelated studies, 470 reviews, 132 studies evaluating the risk stratification but not the diagnostic capacity of echocardiography, 46 studies analyzing patients with shock or persistent hypotension, 41 studies evaluating the diagnostic yield of transesophageal echocardiography in various settings, 18 studies evaluating COVID-19 subjects and 12 studies evaluating the diagnostic yield of echocardiography in settings different from the ED from the analysis. Of the 33 selected studies, 23 were excluded for pre-specified reasons (non-English language, small sample size, patients’ selection, missing data for analysis and use of non-conventional echocardiographic methods). We synthesized the paper selection process in the flow diagram (Figure 1). 10 papers were deemed suitable for further analyses, as synthesized in Figure 1 and Supplementary Materials Table S2. The QUADAS-2 results are synthesized in Supplementary Materials Table S3. Among the selected works, we observed that the most commonly searched signs in the ED were (i) RV dilatation (9 papers, 1025 participants); (ii) McConnell’s sign (4 papers, 506 participants); (iii) tricuspid regurgitant velocity (6 papers, 626 participants); (iv) paradoxical septum movement (6 papers, 713 participants) and (v) reduced RV contractility, expressed by reduced tricuspid anulus plane systolic excursion (TAPSE) (4 papers, 529 participants). The main bias, present in almost all of the studies, is represented by different reference standards for PE diagnosis between patients in the same study and among different studies. This is due to both patients’ characteristics and the different year of execution of each study; most recent studies prefer to adopt CTPA as a reference standard, while temporally distant papers used pulmonary angiography or V/Q scan. However, these tests reflect the common clinical practice in PE and did not affect the results of our study. Other observed biases were: (i) the presence of different cutoffs adopted for certain measurements, such as tricuspid regurgitant velocity or right ventricle end-diastolic diameter; (ii) the varying definitions of right ventricle dilatation across the different studies (RV end-diastolic diameter in parasternal long-axis view [12,13,14,15]; RV/LV ratio in apical four chambers view [12,16,17,18,19]; RV end-diastolic diameter in apical four chambers view [13,15,17,20]) and (iii) the lack of a uniform assessment of pre-test probability in the selected studies, ranging from no assessment to physician’s gestalt to a Wells’ score assessment, which is reflected in the fluctuation in the prevalence of PE throughout the selected studies (iv) the presence in two selected studies of both ED patients and inpatients [16,17]. However, removing these studies from our analysis did not affect our results (data not shown).

**Figure 2:**
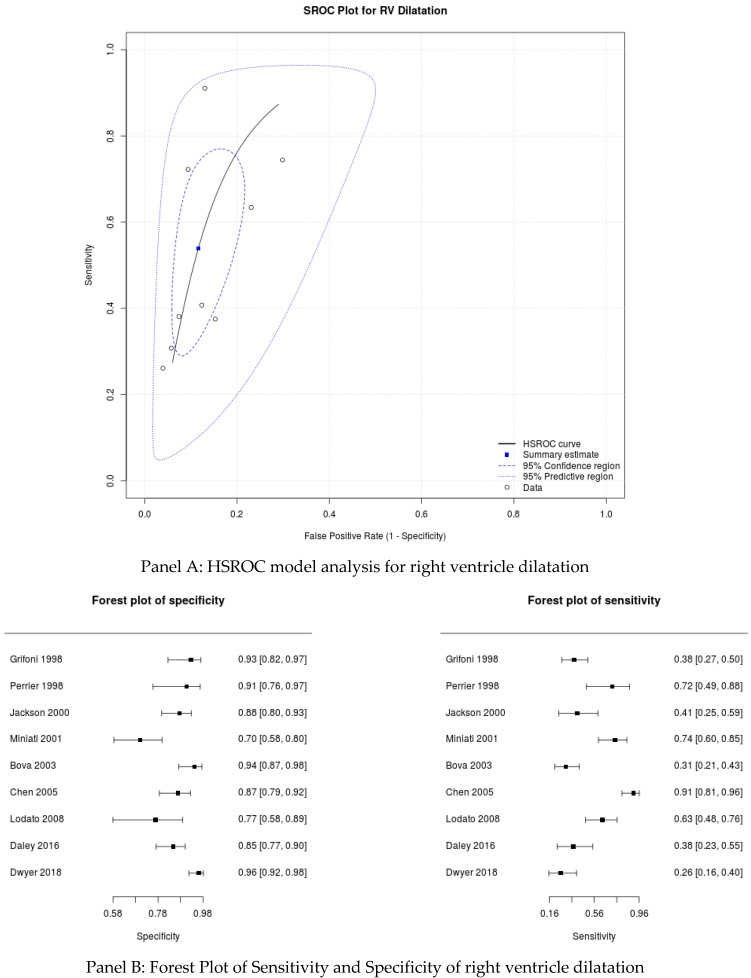
HSROC model and Forest Plot for right ventricle dilatation.

**Figure 3:**
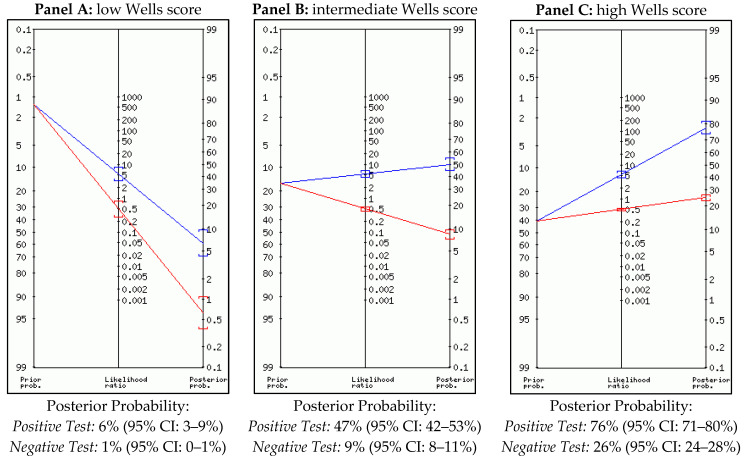
Fagan plot for right ventricle dilatation.

Right ventricle dilatation: the forest plot for RV dilatation and the HSROC model are shown in Figure 2, and the sensitivity analysis is synthesized in Table 2. The post-test modification, according to Wells’ pre-test stratification in accordance with RV dilatation, is synthesized in Figure 3.

McConnell’s Sign: the HSROC model and the forest plot for McConnell’s sign are shown in Supplementary Materials Figure S1, and the sensitivity analysis is shown in Table 2. The post-test modification, according to Wells’ pre-test stratification in accordance with McConnell’s sign, is synthesized in Supplementary Materials Figure S2.

Tricuspid regurgitant velocity: the forest plot for tricuspid regurgitant velocity and the HSROC model are shown in Supplementary Materials Figure S3, the sensitivity analysis is shown in Table 2. The post-test modification according to Wells’ pre-test stratification in accordance with tricuspid regurgitant velocity is synthesized in Supplementary Materials Figure S4.

Paradoxical septum movement: the forest plot for paradoxical septum movement and the HSROC model are shown in Supplementary Materials Figure S5 and the sensitivity analysis is displayed in Table 2. The post-test modification according to Wells’ pre-test stratification in accordance with paradoxical septum movement is synthesized in Supplementary Materials Figure S6.

Reduced right ventricle contractility: the forest plot for reduced RV contractility and the HSROC model are shown in Supplementary Materials Figure S7 and the sensitivity analysis in Table 2. The post-test modification according to Wells’ pre-test stratification in accordance with reduced RV contractility is synthesized in Supplementary Materials Figure S8.

## 4. Discussion

The increasing availability of bedside ultrasound imaging, especially in the ED, is allowing physicians to obtain faster and better assessments of the patient’s hemodynamic status [22]. This technique is particularly useful in patients affected by shock, as it helps the physician to perform a fast differential diagnosis, guide the treatment of specific causes and monitor the patient’s hemodynamics until stabilization [23,24]. According to ESC guidelines, bedside echocardiography can guide fibrinolysis in ED subjects presenting shock, cardiac arrest or persistent hypotension with signs of acute RV overload in the suspect of massive PE [5]. This approach is both specific and sensitive, and can be further improved by extending the ultrasound examination to the deep veins of a patient’s lower limbs [25]. In the remaining outpatients affected by hemodynamically stable PE, the diagnosis should be based on stratification with clinical prediction rulers such as Wells’ score, followed by d-Dimer testing and/or CTPA [26]. However, several papers in which studies were performed different clinical settings underlined a relatively high specificity of different echocardiographic signs, suggesting the use of cardiac ultrasound can support PE diagnosis, even in hemodynamically stable patients [6]. In this paper we aim to underline the importance of applying a clinical method by stratifying the pretest probability in hemodynamically stable patients before the echocardiographic examination. Our results, derived from cohorts of subjects admitted to the ED, underline the unusefulness of performing a cardiac ultrasound if the pretest probability is low or intermediate, independently of echocardiographic results. In fact, the absence or the presence of the analyzed echocardiographic signs at low- or intermediate- pretest probability do not significantly modify the post-test probability and can thus be misleading for the emergency physician. Several authors underlined that McConnell’s sign can be mimicked by right ventricle infarction [27] or can be found in several types of chronic pulmonary hypertension [28]; this is only one of the potential hazards in substituting clinical reasoning with bedside imaging. The application of cardiac ultrasound examination without a prior clinical reasoning and a necessary risk stratification could turn into an excess of unnecessary CTPA, which is one of the main problems when treating PE [29]. Thus, patients at low- or intermediate- pretest probability should be assessed clinically with d-Dimer testing, according to international guidelines. A potential field of development is the use of transthoracic echocardiography in patients with a high pretest probability. Among these patients, the presence of any single echocardiographic sign of RV overload could significantly increase the post-test probability at a level sufficient to rationally suspect PE. Thus, among subjects with a high pretest probability, some authors suggest that the application of a multi-organ ultrasound approach (echocardiography, doppler ultrasound and thoracic ultrasound) could improve both sensitivity and specificity [30]. However, in absence of prospectively conducted studies, hemodynamically stable subjects with a high pretest probability for PE should undergo CTPA in order to confirm the clinical suspicion, independently of echocardiographic assessment. Moreover, in patients with a high PE pretest probability, the absence of echocardiographic signs of right ventricle overload do not allow the emergency physician to exclude the presence of PE, since the post-test probability of a negative examination remains too high.

## 5. Study Limitations

The main study limitations are mainly related to (i) a research strategy that was limited to Pubmed/MedLine, Web of Science and Cochrane Database repositories, further implementations of the current review will also comprise the EMBASE repository, (ii) the pre-test probability level remains theoretical; further real-life studies are needed to assess the performance of the echocardiographic signs with different pretest probabilities to validate our observations.

## 6. Conclusions

Despite the advances in the application of bedside ultrasound in the ED, according to our results, echocardiography should not be adopted to modify the clinical behavior of the emergency physician before the diagnosis of hemodynamically stable PE. Patients with a high clinical pretest probability and echocardiographic signs of right ventricle overload will benefit the most from an increase in post-test probability. In this last population, however, it is reasonable to perform further studies to evaluate whether an echocardiographic approach could be helpful to confirm PE diagnosis in the setting of the ED. A focused cardiac and thoracic ultrasound examination, however, is useful in the differential diagnosis of chest pain and dyspnea in an acute setting.

## Figures and Tables

**Table 1:** Wells’ score for pulmonary embolism according to its original definition.

Clinical signs and symptoms for DVT	No = 0 points Yes = +3 points
Pulmonary embolism is the first hypothesis or Pulmonary embolism is equally likely	No = 0 points Yes = +3 points
Heart rate >100 bpm	No = 0 points Yes = +1.5 points
Immobilization at least 3 days or Surgery in the previous 4 weeks	No = 0 points Yes = +1.5 points
Previous, objectively diagnosed pulmonary embolism or Previous, objectively diagnosed deep vein thrombosis	No = 0 points Yes = +1.5 points
Hemoptysis	No = 0 points Yes = +1 point
Malignancy w/treatment within 6 months or palliative	No = 0 points Yes = +1 point

**Table 2:** Sensitivity analysis and diagnostic odds ratio from meta-analysis.

	Se (97.5% CI)	Sp (97.5% CI)	DOR (95% CI)
*RV Dilatation* [12,13,14,15,16,17,18,19,20]	0.539 (0.695)	0.884 (0.925)	8.907 (5.025–15.792)
*McConnell’s Sign* [17,18,19,21]	0.175 (0.240)	0.971 (0.984)	6.985 (3.313–14.727)
*Tricuspid regurgitant velocity* [13,14,15,16,20]	0.473 (0.612)	0.842 (0.922)	4.791 (2.101–10.928)
*Paradoxical septum* [12,15,17,18,19,20]	0.232 (0.299)	0.968 (0.989)	9.080 (3.419–24.113)
*Reduced RV contractility* [15,18,19,20]	0.563 (0.695)	0.843 (0.908)	6.492 (4.390–10.978)

Legend: RV: right ventricle; Se: Sensitivity; Sp: Specificity; DOR: Diagnostic Odds Ratio; CI: confidence interval.

## Data Availability

All data generated or analyzed during this study are included in this published article [and its supplementary information files].

## Supplementary Materials

The following are available online at https://www.mdpi.com/1648-9144/57/8/766/s1, Figure S1: HSROC model and Forest Plot for McConnell’s Sign, Figure S2: Fagan plots for McConnell’s sign; Table S1: Search strategy adopted in the systematic review, Table S2: Characteristics of the selected papers, Table S3: QUADAS-2 assessment table for the selected studies; Figure S3: HSROC model and Forest Plot for tricuspid regurgitant velocity; Figure S4: Fagan plots for tricuspid regurgitant velocity; Figure S5: HSROC model and Forest Plot for paradoxycal septum movement; Figure S6: Fagan plots for paradoxical septum movement; Figure S7: HSROC model and Forest Plot for right ventricle hypokinesis; Figure S8: Fagan plots for Right Ventricle Hypokinesis.

## References

[1] (2017). Ultrasound Guidelines: Emergency, Point-of-Care and Clinical Ultrasound Guidelines in Medicine. Ann. Emerg. Med..

[2] Perrone T., Maggi A., Sgarlata C., Palumbo I., Mossolani E., Ferrari S., Melloul A., Mussinelli R., Boldrini M., Raimondi A. (2017). Lung ultrasound in internal medicine: A bedside help to increase accuracy in the diagnosis of dyspnea. Eur. J. Intern. Med..

[3] Bekgoz B., Kilicaslan I., Bildik F., Keles A., Demircan A., Hakoglu O., Coskun G., Demir H.A. (2019). BLUE protocol ultrasonography in Emergency Department patients presenting with acute dyspnea. Am. J. Emerg. Med..

[4] Wright J., Jarman R., Connolly J., Dissmann P. (2009). Echocardiography in the emergency department. Emerg. Med. J..

[5] Konstantinides S.V., Meyer G., Becattini C., Bueno H., Geersing G.-J., Harjola V.-P. (2019). 2019 ESC Guidelines for the diagnosis and management of acute pulmonary embolism developed in collaboration with the European Respiratory Society (ERS): The Task Force for the diagnosis and management of acute pulmonary embolism of the European Society of Cardiology (ESC). Eur. Heart J..

[6] Fields J.M., Davis J., Girson L., Au A., Potts J., Morgan C.J., Vetter I., Riesenberg L.A. (2017). Transthoracic Echocardiography for Diagnosing Pulmonary Embolism: A Systematic Review and Meta-Analysis. J. Am. Soc. Echocardiogr..

[7] Moher D., Liberati A., Tetzlaff J., Altman D.G., Altman D. (2009). Preferred Reporting Items for Systematic Reviews and Meta-Analyses: The PRISMA Statement. PLoS Med..

[8] Whiting P., Rutjes A.W., Reitsma J.B., Bossuyt P.M., Kleijnen J. (2003). The development of QUADAS: A tool for the quality assessment of studies of diagnostic accuracy included in systematic reviews. BMC Med. Res. Methodol..

[9] Patel A., Cooper N., Freeman S., Sutton A. (2021). Graphical enhancements to summary receiver operating characteristic plots to facilitate the analysis and reporting of meta-analysis of diagnostic test accuracy data. Res. Synth. Methods.

[10] Wells P.S., Anderson D.R., Rodger M., Stiell I., Dreyer J.F., Barnes D., Forgie M., Kovacs G., Ward J., Kovacs M.J. (2001). Excluding pulmonary embolism at the bedside without diagnostic imaging: Management of patients with suspected pulmonary embolism presenting to the emergency department by using a simple clinical model and d-dimer. Ann. Intern. Med..

[11] Schwartz A. (2006). Diagnostic Test Calculator (version 2010042101). http://ulan.mede.uic.edu/cgi-bin/testcalc.pl.

[12] Grifoni S., Olivotto I., Cecchini P., Pieralli F., Camaiti A., Santoro G., Pieri A., Toccafondi S., Magazzini S., Berni G. (1998). Utility of an integrated clinical, echocardiographic, and venous ultrasonographic approach for triage of patients with suspected pulmonary embolism. Am. J. Cardiol..

[13] Perrier A., Tamm C., Unger P.-F., Lerch R., Sztajzel J. (1998). Diagnostic accuracy of Doppler-echocardiography in unselected patients with suspected pulmonary embolism. Int. J. Cardiol..

[14] Miniati M., Monti S., Pratali L., Di Ricco G., Marini C., Formichi B., Prediletto R., Michelassi C., Di Lorenzo M., Tonelli L. (2001). Value of transthoracic echocardiography in the diagnosis of pulmonary embolism: Results of a prospective study in unselected patients. Am. J. Med..

[15] Chen J.-Y., Chao T.-H., Guo Y.-L., Hsu C.-H., Huang Y.-Y., Chen J.-H., Lin L.J. (2006). A Simplified Clinical Model to Predict Pulmonary Embolism in Patients With Acute Dyspnea. Int. Heart J..

[16] Bova C., Greco F., Misuraca G., Serafini O., Crocco F., Greco A., Noto A. (2003). Diagnostic utility of echocardiography in patients with suspected pulmonary embolism. Am. J. Emerg. Med..

[17] Lodato J.A., Ward R.P., Lang R.M. (2008). Echocardiographic Predictors of Pulmonary Embolism in Patients Referred for Helical CT. Echocardiography.

[18] Daley J., Grotberg J., Pare J., Medoro A., Liu R., Hall M.K., Taylor A., Moore C.L. (2017). Emergency physician performed tricuspid annular plane systolic excursion in the evaluation of suspected pulmonary embolism. Am. J. Emerg. Med..

[19] Dwyer K.H., Rempell J.S., Stone M.B. (2018). Diagnosing centrally located pulmonary embolisms in the emergency department using point-of-care ultrasound. Am. J. Emerg. Med..

[20] Jackson R.E., Rudoni R.R., Hauser A.M., Pascual R.G., Hussey M.E. (2000). Prospective Evaluation of Two-dimensional Transthoracic Echocardiography in Emergency Department Patients with Suspected Pulmonary Embolism. Acad. Emerg. Med..

[21] Kalkan A.K., Ozturk D., Erturk M., Kalkan M.E., Cakmak H.A., Oner E., Uzun F., Tasbulak O., Yakisan T., Celik A. (2016). The diagnostic value of serum copeptin levels in an acute pulmonary embolism. Cardiol. J..

[22] Whitson M.R., Mayo P.H. (2016). Ultrasonography in the emergency department. Crit. Care.

[23] Wacker D.A., Winters M.E. (2014). Shock. Emerg. Med. Clin. N. Am..

[24] Shrestha G.S., Srinivasan S. (2018). Role of Point-of-Care Ultrasonography for the Management of Sepsis and Septic Shock. Rev. Recent Clin. Trials.

[25] Nazerian P., Volpicelli G., Gigli C., Lamorte A., Grifoni S., Vanni S. (2018). Diagnostic accuracy of focused cardiac and venous ultrasound examinations in patients with shock and suspected pulmonary embolism. Intern. Emerg. Med..

[26] Konstantinides S.V., Meyer G. (2019). The 2019 ESC Guidelines on the Diagnosis and Management of Acute Pulmonary Embolism. Eur. Heart J..

[27] Casazza F., Bongarzoni A., Capozi A., Agostoni O. (2005). Regional right ventricular dysfunction in acute pulmonary embolism and right ventricular infarction. Eur. J. Echocardiogr..

[28] Walsh B.M., Moore C.L. (2015). McConnell’s Sign Is Not Specific for Pulmonary Embolism: Case Report and Review of the Literature. J. Emerg. Med..

[29] Molaee S., Ghanaati H., Safavi E., Foroumandi M., Peiman S. (2015). Computed Tomography Pulmonary Angiography for Evaluation of Patients with Suspected Pulmonary Embolism: Use or Overuse. Iran J. Radiol..

[30] Nazerian P., Vanni S., Volpicelli G., Gigli C., Zanobetti M., Bartolucci M., Ciavattone A., Lamorte A., Veltri A., Fabbri A. (2014). Accuracy of Point-of-Care Multiorgan Ultrasonography for the Diagnosis of Pulmonary Embolism. Chest.

