# Supplementary material for: Clinical Method Applied to Focused Ultrasound: The Case of Wells’ Score and Echocardiography in the Emergency Department: A Systematic Review and a Meta-Analysis
Source: Medicina (Kaunas). 2021 Jul 28;57(8):766. doi: 10.3390/medicina57080766 (PMC8400535; doi:10.3390/medicina57080766)
Supplement: Supplementary file 1 [file medicina-57-00766-s001.zip › medicina-1258730-supplementary.pdf]

Clinical method applied to focused ultrasound: the case of Wells' score and echocardiography in the emergency department: a systematic review and a meta-analysis.

Supplementary Online-Only Material

Table S1: Search strategy adopted in the systematic review.

Research String	*Database*	*Retrieved Papers*	*Selected Papers*
(pulmonary embolism[MeSH Major Topic]) AND (echocardiography[MeSH Major Topic])	PubMed	565	Bova 2003
(pulmonary embolism[MeSH Major Topic]) AND ("echocardiography, doppler"[MeSH Major Topic])	PubMed	76	Perrier 1998
("pulmonary embolism/diagnosis"[MeSH Major Topic]) AND ("echocardiography"[MeSH Major Topic])	PubMed	441	Daley 2016<br>Bova 2003<br>Perrier 1998
("echocardiography, transthoracic"[MeSH Terms]) AND ("pulmonary embolism[MeSH Terms])	PubMed	2219	Daley 2016<br>Kalkan 2016<br>Lodato 2008<br>Chen 2005<br>Bova 2003<br>Miniati 2001<br>Grifoni 1998<br>Perrier 1998
"pulmonary embolism"[Topic] AND "echocardiography"[Topic] AND "diagnosis" [Topic] and "emergency department"[Topic]	WoS	131	Daley 2016
("pulmonary embolism"[Topic]) AND ("echocardiography"[Topic])	WoS	2039	Dwyer 2018<br>Daley 2016<br>Lodato 2008

			Chen 2005<br><br>Bova 2003<br><br>Miniati 2001<br><br>Grifoni 1998<br><br>Perrier 1998

Table S2: Characteristics of the selected papers

Name and Year	Design	Patients	Diagnosis	N	RV Dilatation			
					TP	FP	TN	FN
Grifoni 1998[1]	Cohort	ED	V/Q scan Angiography	117	24	4	50	39
Perrier 1998[2]	Cohort	ED	V/Q scan	50	13	3	29	5
Jackson 2000[3]	Prospective cohort	ED	V/Q scan Angiography CTPA	124	11	12	85	16
Miniati 2001[4]	Cohort	ED	V/Q scan Angiography	110	32	20	47	11
Bova 2003[5]	Prospective cohort	ED and Inpatients	V/Q scan Angiography	152	20	5	82	45
Chen 2005[6]	Retrospective cohort	ED	V/Q scan Angiography	148	51	12	80	5
Lodato 2008[7]	Retrospective cohort	ED and Inpatients	CTPA	67	26	6	20	15
Kalkan 2016[8]	Prospective cohort	ED	CTPA	90	--	--	--	--
Daley 2016[9]	Prospective cohort	ED	CTPA	150	12	18	20	100
Dwyer 2018[10]	Prospective cohort	ED	CTPA	199	11	6	147	35

Table S3: QUADAS-2 assessment table for the selected studies

**Domain #1: Patients Selection**	**Was a consecutive or random sample of patients enrolled?**	**Was a case/control study avoided ?**	**Did the study avoid inappropriate exclusions ?**	**Could the patients' selection represent a potential source of bias ?**	**Are there concerns that the included patients do not match the review question?**	**Describe methods of patient selection: Describe included patients (prior testing, presentation, intended use of index test and setting)**
	**yes/no/unclear**	**yes/no/unclear**	**yes/no/unclear**	**yes/no/unclear**	**yes/no/unclear**	
Grifoni 1998	yes	Yes	yes	Yes	no	outpatients with suspect PE; no prior testing; haemodynamically stable (110/117) and unstable (7/117); clinical evaluation and echocardiography before V/Q scan and angiography; setting: ED
Perrier 1998	yes	Yes	yes	Yes	no	outpatients with suspect PE; no prior testing; haemodynamically stable (50/50); clinical evaluation before examination; echocardiography before V/Q scan and angiography; setting: ED (48/50)
Jackson 2000	yes	Yes	yes	No	no	outpatients with suspect PE; no prior testing; haemodynamically stable; clinical evaluation before examination; echocardiography performed before or ordered by ED physician with second level examinations (lung angiography; V/Q scan, CTPA, lower limb ultrasound); setting: ED
Miniati 2001	yes	Yes	unclear	Unclear	no	outpatients with supect PE; no prior testing; haemodynamically stable; clinical evaluation and echocardiography before V/Q scan and angiography; setting: ED (evaluation by pneumologists)
Bova 2003	yes	Yes	unclear	No	yes	outpatients (151) and inpatients (101) with suspect PE; clinical symptoms compatible with PE; echocardiography performed during the diagnostic work-up; setting: ED and inpatients
Chen 2005	yes	No	yes	No	no	outpatients with dyspnea; no prior testing; haemodynamically stable; echocardiography before V/Q scan and angiography; setting: ED
Lodato 2008	yes	Yes	yes	No	no	patients undergoing to CT scan and evaluated with echocardiography within 48 hours from CT scan
Kalkan 2016	Yes	Yes	unclear	No	no	outpatients with suspect PE (chest pain or dyspnoea); haemodynamically stable; clinical evaluation followed by CTPA and echocardiography; setting: ED
Daley 2016	unclear	Yes	yes	Yes	no	outpatients with suspect PE; haemodynamically stable (30/32) and unstable (2/32); echocardiography ordered once CTPA is asked by the attending physician; setting: ED
Dwyer 2018	yes	Yes	yes	Unclear	no	outpatients with suspect PE; central and peripheral PE; of the central PE, 6/20 required rTPA; echocardiography and lower limb ultrasound ordered once CTPA was asked by the attending physician; setting: ED
**Domain #2: Index Test**	**Were the index test results interpreted without knowledge of the results of the reference standard?**	**If a threshold was used, was it pre-specified?**	**Could the conduct or interpretation of the index test have introduced bias?**	**Are there concerns that the index test, its conduct, or interpretation differ from the review question?**		**Describe the index test and how it was conducted and interpreted**
	**yes/no/unclear**	**yes/no/unclear**	**high/low/unclear**	**yes/no/unclear**		
Grifoni 1998	yes	Yes	low	No		echocardiography and limb vein ultrasound performed after clinical evaluation and before second-level examinations (V/Q scan and angiography)
Perrier 1998	yes	Unclear	low	No		echocardiography performed after clinical evaluation and before second-level examinations; the cutoff for tricuspid regurgitant velocity is not prespecified

Jackson 2000	yes	Yes	low	No		echocardiography performed before or during the diagnostic workup to confirm PE
Miniati 2001	yes	No	low	No		echocardiography within 2 hours from the first evaluation
Bova 2003	yes	Yes	low	No		echocardiography in the setting of a diagnostic workup for PE
Chen 2005	yes	Yes	high	No		echocardiography in the setting of a diagnostic workup for PE
Lodato 2008	yes	Yes	unclear	No		echocardiography after CTPA for suspected PE
Kalkan 2016	unclear	Yes	low	No		echocardiography in the setting of a diagnostic workup for PE; intermediate or high pre-test probability assessed with Wells score.
Daley 2016	yes	Yes	low	No		echocardiography before second-level imaging; two TAPSE cutoff (17 and 20 mm) tested.
Dwyer 2018	yes	Yes	low	No		echocardiography in patients undergoing CTPA for suspect PE
**Domain #3: Reference Standard**	**Is the reference standard likely to correctly classify the target condition?**	**Were the reference standard results interpreted without knowledge of the results of the index test?**	**Could the reference standard, its conduct, or its interpretation have introduced bias?**	**Are there concerns that the target condition as defined by the reference standard does not match the review question?**		**Describe the reference standard and how it was conducted and interpreted**
	**yes/no/unclear**	**yes/no/unclear**	**low/high/unclear**	**low/high/unclear**		
Grifoni 1998	yes	Yes	low	Low		V/Q scan; Pulmonary Angiography; Lower Limb Ultrasoiund; Evaluation of Pre-Test probability at Triage.
Perrier 1998	yes	Yes	low	Low		V/Q scan; Pulmonary Angiography; Lower Limb Ultrasound; Evaluation of Pre-Test probability before exam.
Jackson 2000	yes	Yes	low	Low		V/Q scan; CTPA; Lower Limb Ultrasound ; Pulmonary Angiography, MRA. Evaluation of Pre-Test probability according to physician's "clinical gestalt".
Miniati 2001	yes	Yes	low	Low		V/Q scan; Pulmonary Angiography; Lower Limb Ultrasoiund; Evaluation of Pre-Test probability before exam.
Bova 2003	yes	Yes	low	Low		V/Q scan; Pulmonary Angiography during the diagnostic workup for suspected PE (high risk).
Chen 2005	yes	Unclear	low	Low		V/Q scan; Pulmonary Angiography; Evaluation of Pre-Test probability before exam.
Lodato 2008	yes	Yes	low	Low		CTPA in the diagnostic workup for PE
Kalkan 2016	yes	Unclear	low	Low		CTPA in patients with intermediate or high pretest probability
Daley 2016	yes	Yes	low	Low		CTPA ordered by the attending physician
Dwyer 2018	yes	Yes	low	Low		CTPA ordered by the attending physician
**Domain #4: Flow and Timing**	**Was there an appropriate interval between index test(s) and reference standard?**	**Did all patients receive a reference standard?**	**Did all patients receive the same reference standard?**	**Were all patients included in the analysis?**	**Could the patient flow have introduced bias?**	**Describe any patients who did not receive the index test(s) and/or reference standard or who were excluded from the 2x2 table (refer to flow diagram). Describe the time interval and any interventions between index test(s) and reference standard.**
	**yes/no/unclear**	**yes/no/unclear**	**yes/no/unclear**	**yes/no/unclear**	**low/high/unclear**	
Grifoni 1998	yes	Yes	no	Yes	low	No patients excluded from the 2x2 table; echocardiography performed before the second-level examination.
Perrier 1998	yes	Yes	no	Yes	low	No patients excluded from the 2x2 table; echocardiography performed 14 hours after symptoms' onset
Jackson 2000	unclear	Yes	no	Yes	unclear	No patients excluded from the 2x2 table; echocardiography performed before the index text.

Miniati 2001	yes	Yes	no	Yes	low	No patients excluded from the 2x2 table; echocardiography performed before the index text, within 2 hours from the study admission.
Bova 2003	unclear	no	no	No	high	38 patients excluded from the 2x2 table; echocardiography performed in the context of a diagnostic workup for PE
Chen 2005	unclear	Yes	no	Yes	unclear	No patients excluded from the 2x2 table; echocardiography performed in the context of a diagnostic workup for PE
Lodato 2008	No	Yes	yes	Yes	low	Echocardiography performed 48 hours before second-level imaging
Kalkan 2016	unclear	Yes	yes	Yes	low	No patients excluded from the 2x2 table; Echocardiography performed before the second-level imaging in the ED
Daley 2016	Yes	Yes	no	Yes	no	No patients excluded from the 2x2 table; Echocardiography performed before the second-level imaging in the ED
Dwyer 2018	Yes	Yes	no	No	low	9 patients excluded from the 2x2 table; Echocardiography performed before the second-level imaging in the ED

Figure S1: HSROC model and Forest Plot for McConnell's Sign

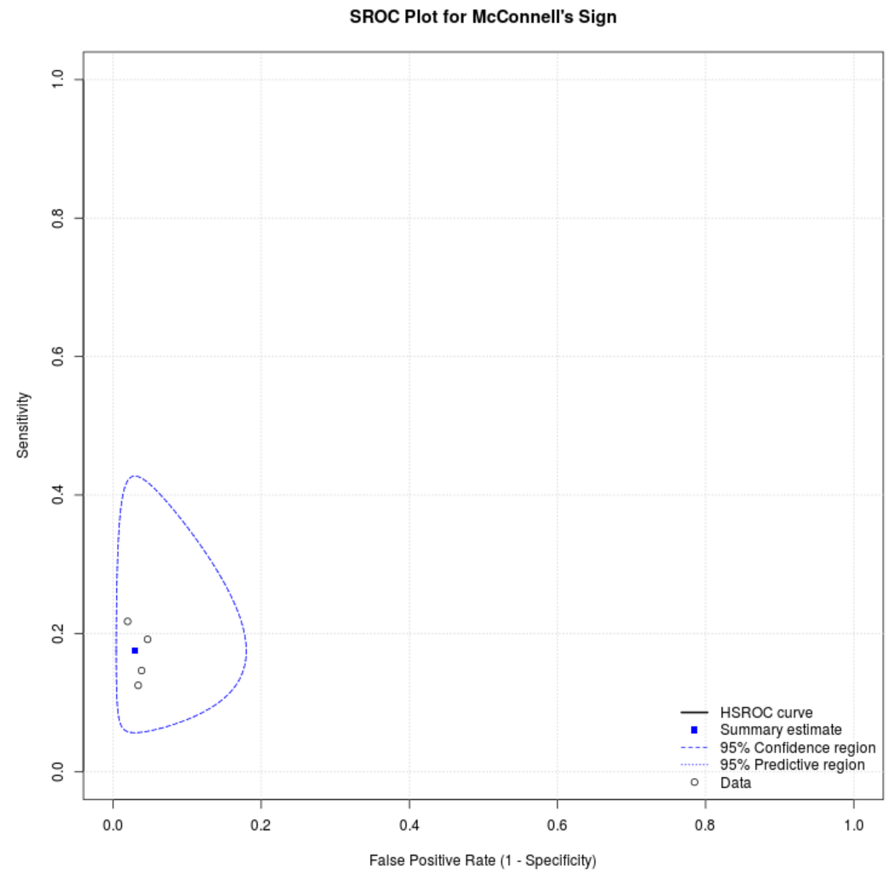

Panel A: HSROC model analysis for McConnell's sign

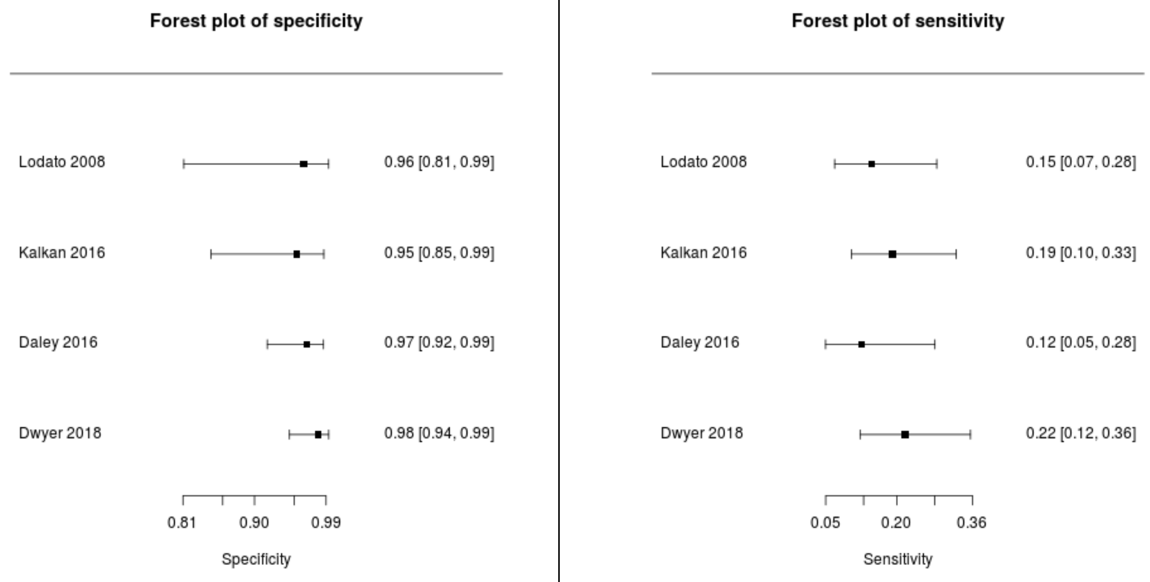

Panel B: Forest Plot of Sensitivity and Specificity of McConnell's sign

Figure S2: Fagan plots for McConnell's sign

<table>
<tr><td>Panel A: low Wells score</td><td>Panel B: intermediate Wells score</td><td>Panel C: high Wells score</td></tr>
<tr><td>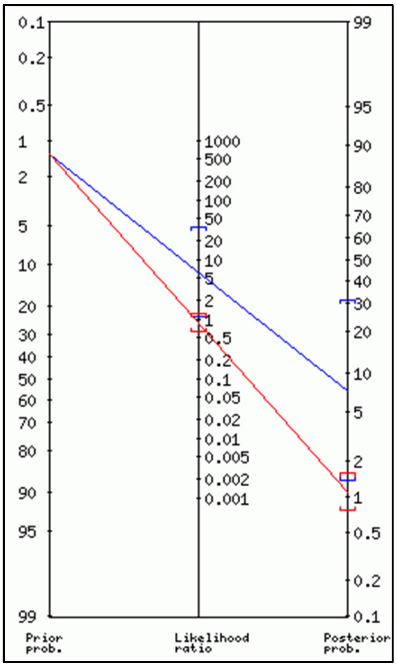
</td><td>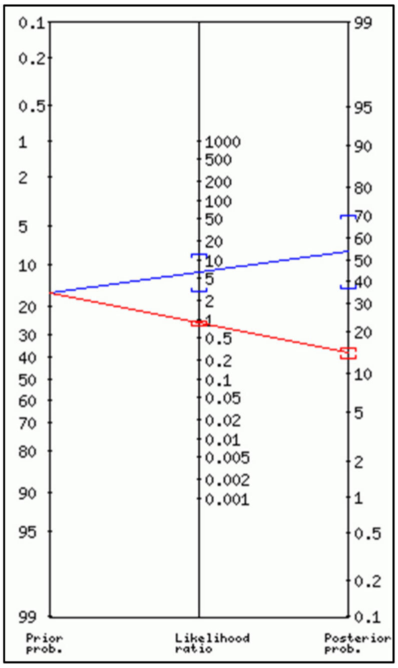
</td><td>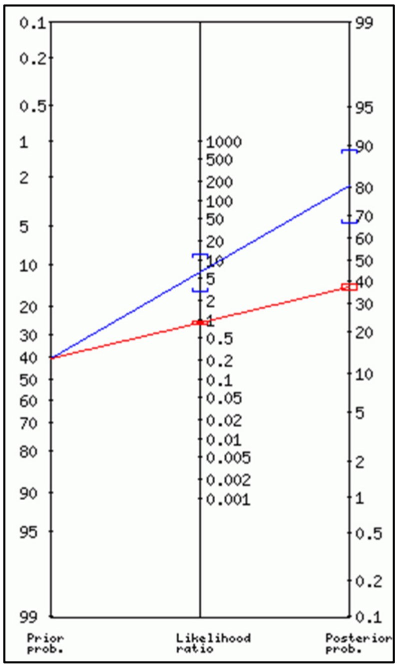
</td></tr>
<tr><td>Posterior Probability:
Positive Test: 7% (95%CI:1-31%)
Negative Test: 1% (95%CI:1-2%)</td><td>Posterior Probability:
Positive Test: 54% (95%CI:36-70%)
Negative Test: 14% (95%CI:13-15%)</td><td>Posterior Probability:
Positive Test: 80% (95%CI:67-89%)
Negative Test: 37% (95%CI:35-38%)</td></tr>
</table>

Figure S3: HSROC model and Forest Plot for tricuspid regurgitant velocity

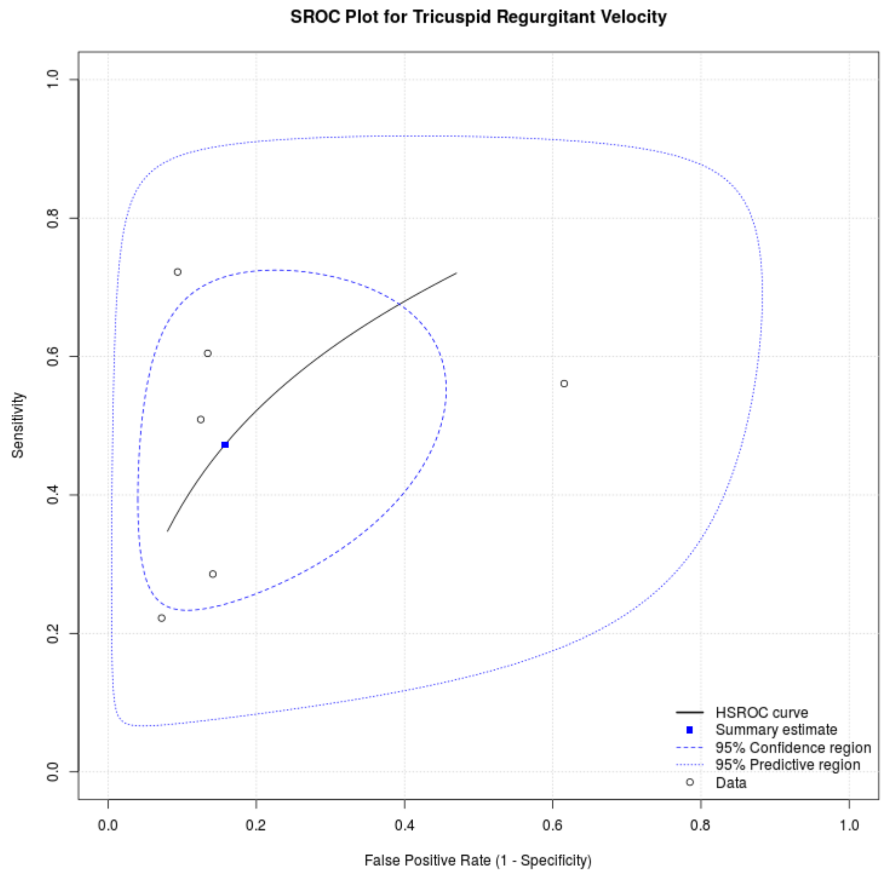

Panel A: HSROC model analysis for Tricuspid Regurgitant Velocity

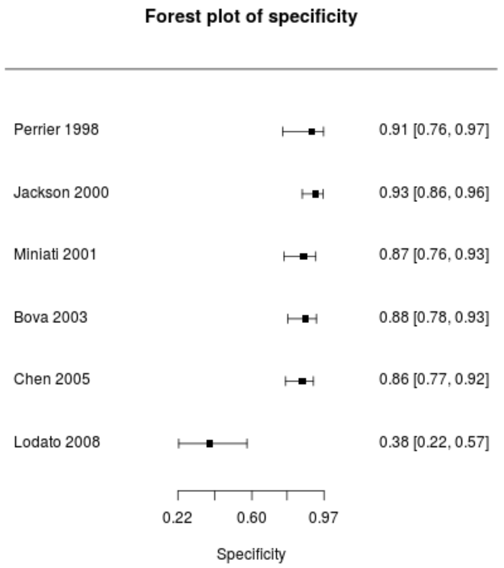

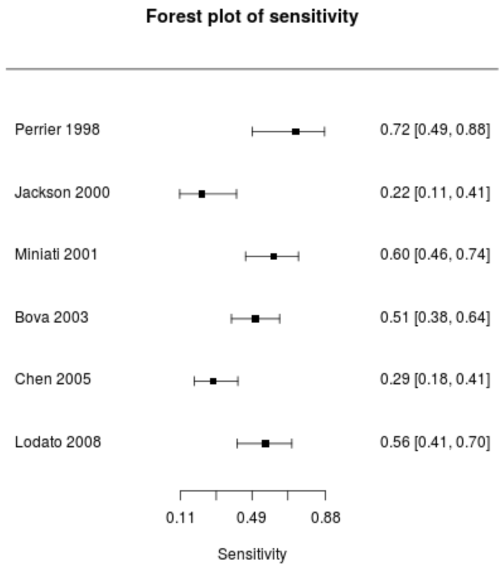

Panel B: Forest Plot of Sensitivity and Specificity of Tricuspid Regurgitant Velocity

Figure S4: Fagan plots for tricuspid regurgitant velocity

<table>
<tr><td>Panel A: low Wells score</td><td>Panel B: intermediate Wells score</td><td>Panel C: high Wells score</td></tr>
<tr><td>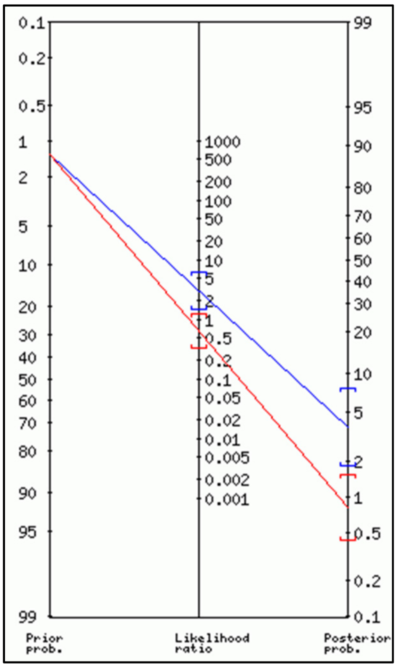</td><td>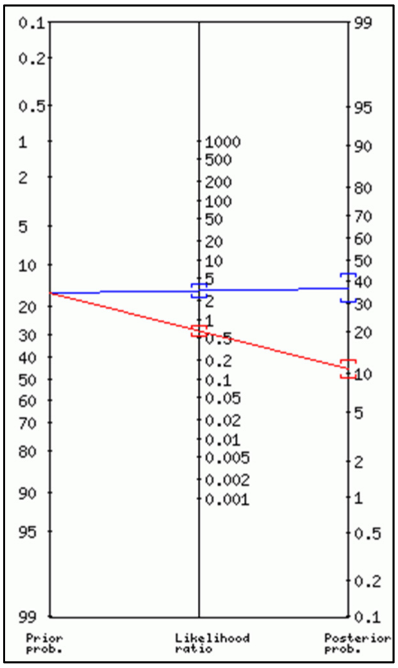</td><td>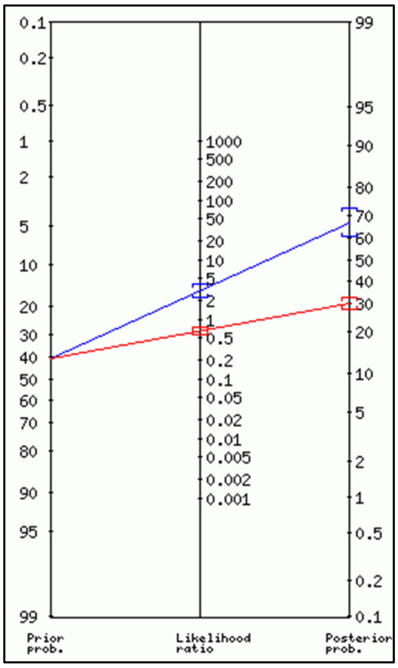</td></tr>
<tr><td>Posterior Probability:

Positive Test: 4% (95%CI: 2-8%)

Negative Test: 1% (95%CI:0-2%)</td><td>Posterior Probability:

Positive Test: 37% (95%CI:30-43%)

Negative Test: 11% (95%CI:9-13%)</td><td>Posterior Probability:

Positive Test: 67% (95%CI:61-73%)

Negative Test: 30% (95%CI:28-33%)</td></tr>
</table>

Figure S5: HSROC model and Forest Plot for paradoxycal septum movement

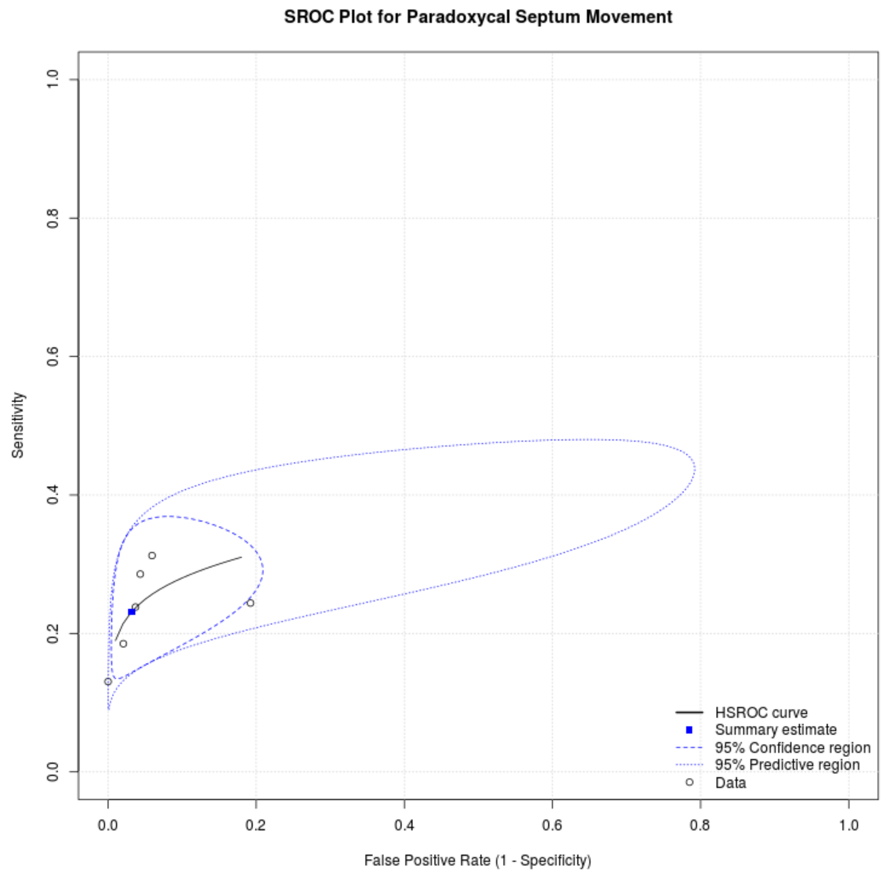

Panel A: HSROC model analysis for Paradoxycal Septum Movement

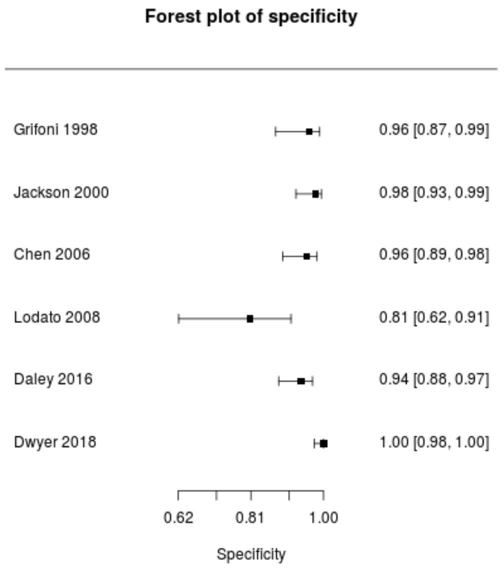

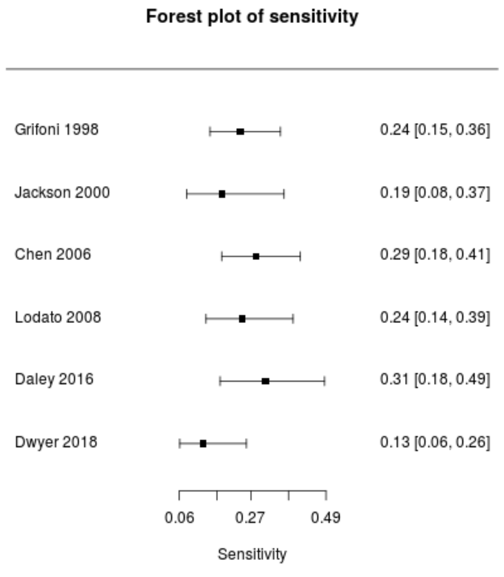

Panel B: Forest Plot of Sensitivity and Specificity of Paradoxycal Septum Movement

Figure S6: Fagan plots for paradoxical septum movement

<table>
<tr>
<td>Panel A: low Wells score</td>
<td>Panel B: intermediate Wells score</td>
<td>Panel C: high Wells score</td>
</tr>
<tr>
<td>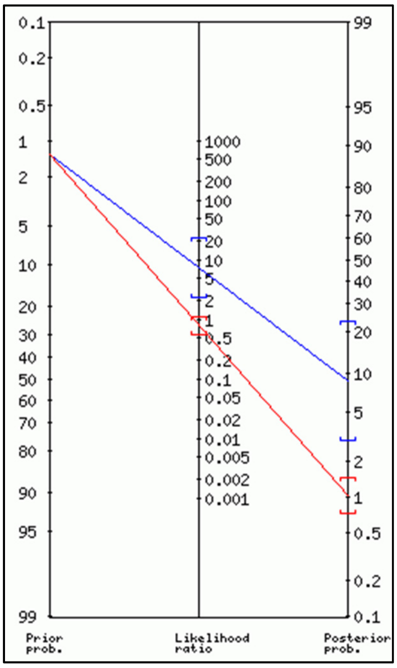
</td>
<td>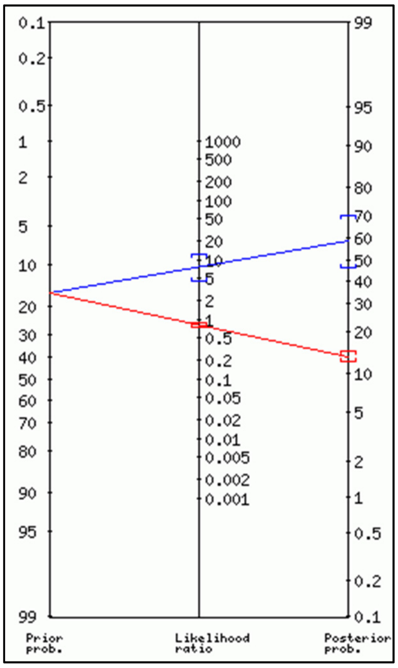
</td>
<td>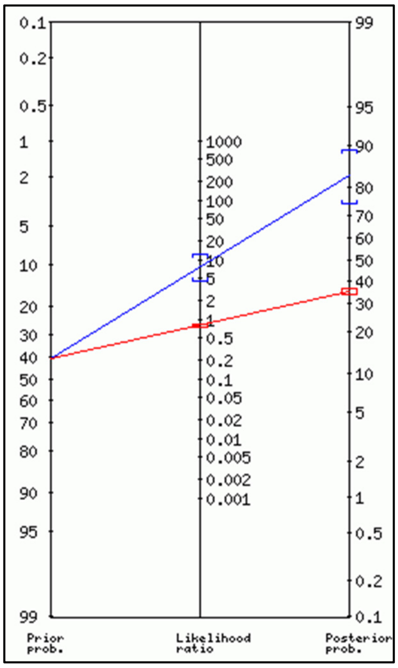
</td>
</tr>
<tr>
<td>Posterior Probability:
Positive Test: 9% (95%CI:3-22%)
Negative Test: 1% (95%CI:0-1%)</td>
<td>Posterior Probability:
Positive Test: 58% (95%CI:47-70%)
Negative Test: 13% (95%CI:12-14%)</td>
<td>Posterior Probability:
Positive Test: 83% (95%CI:76-89%)
Negative Test: 35% (95%CI:34-36%)</td>
</tr>
</table>

Figure S7: HSROC model and Forest Plot for right ventricle hypokinesis

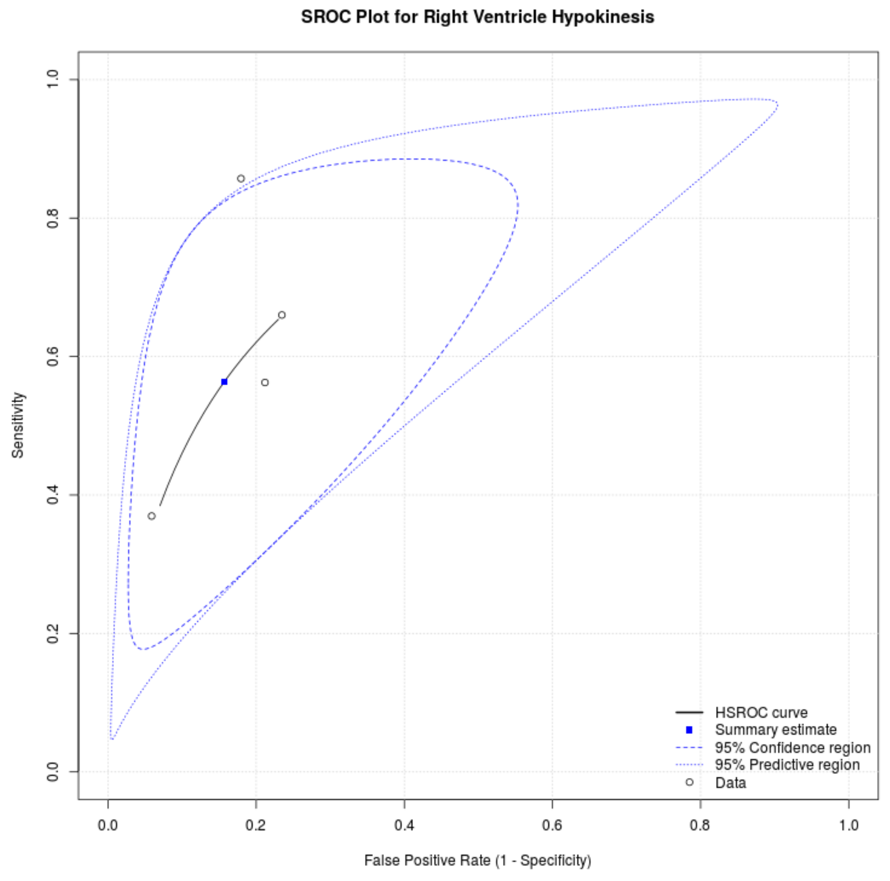

Panel A: HSROC model analysis for Right Ventricle Hypokinesis

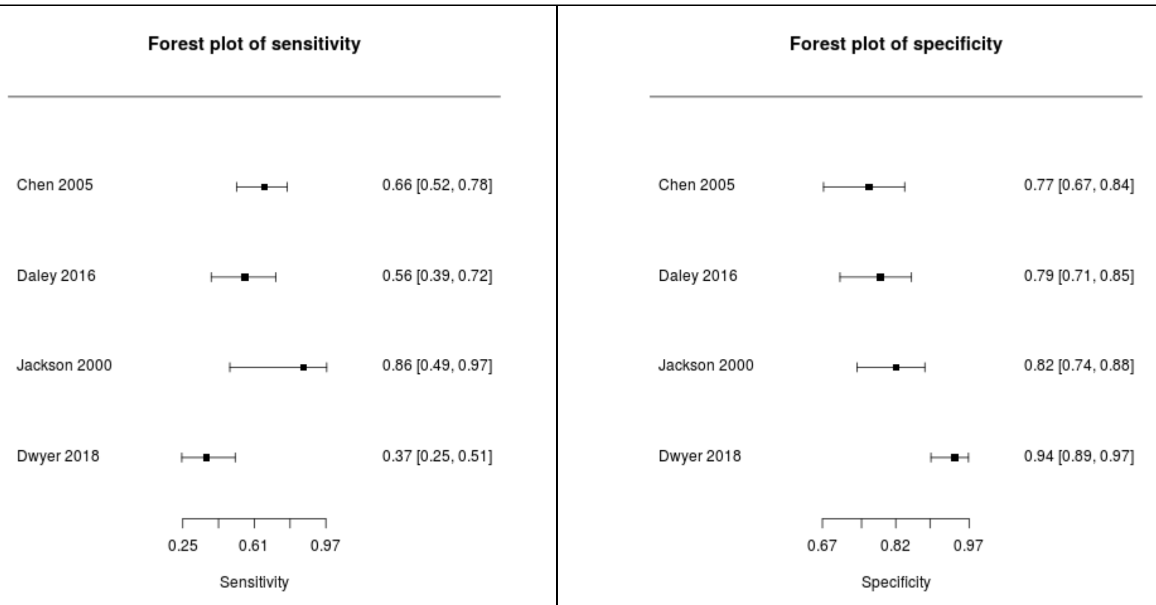

Panel B: Forest Plot of Sensitivity and Specificity of Right Ventricle Hypokinesis

Figure S8: Fagan plots for Right Ventricle Hypokinesis

<table>
<tr><td>Panel A: low Wells score</td><td>Panel B: intermediate Wells score</td><td>Panel C: high Wells score</td></tr>
<tr><td>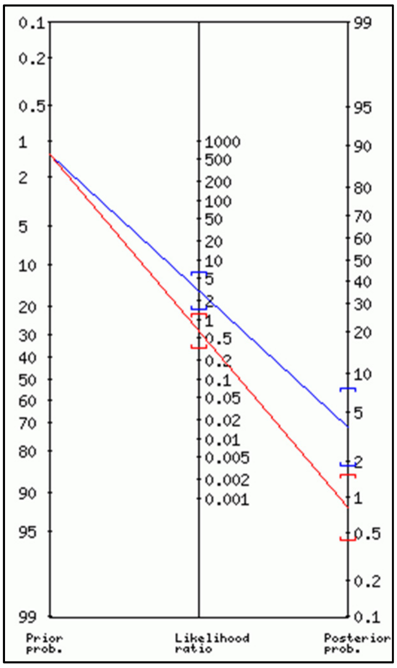
</td><td>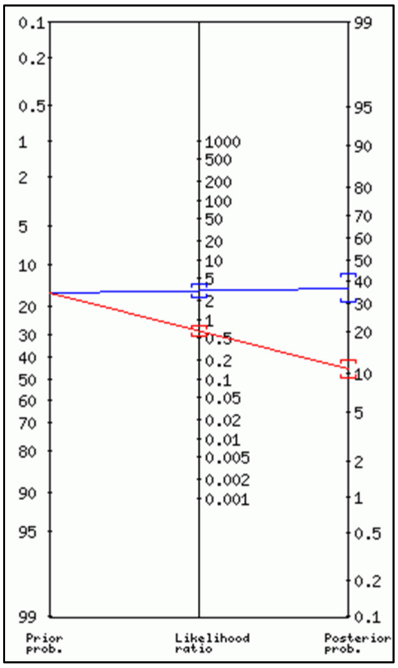
</td><td>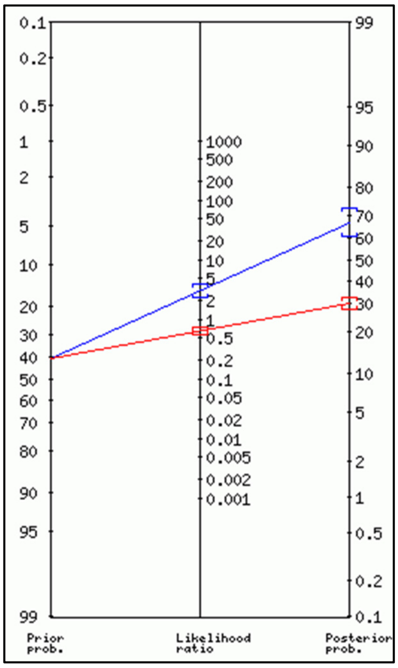
</td></tr>
<tr><td>Posterior Probability:
Positive Test: 5% (95%CI:3-7%)
Negative Test: 1% (95%CI:0-1%)</td><td>Posterior Probability:
Positive Test: 41% (95%CI:36-46%)
Negative Test: 9% (95%CI:8-11%)</td><td>Posterior Probability:
Positive Test: 71% (95%CI:67-75%)
Negative Test: 26% (95%CI:24-28%)</td></tr>
</table>

eReferences

[1] Grifoni S, Olivotto I, Cecchini P, Pieralli F, Camaiti A, Santoro G, et al. Utility of an integrated clinical, echocardiographic, and venous ultrasonographic approach for triage of patients with suspected pulmonary embolism. Am J Cardiol 1998;82:1230–5. doi:10.1016/S0002-9149(98)00612-2.

[2] Perrier A, Tamm C, Unger P-F, Lerch R, Sztajzel J. Diagnostic accuracy of Doppler-echocardiography in unselected patients with suspected pulmonary embolism. Int J Cardiol 1998;65:101–9. doi:10.1016/S0167-5273(98)00107-7.

[3] Jackson RE, Rudoni RR, Hauser AM, Pascual RG, Hussey ME. Prospective Evaluation of Two-dimensional Transthoracic Echocardiography in Emergency Department Patients with Suspected Pulmonary Embolism. Acad Emerg Med 2000;7:994–8. doi:10.1111/j.1553-2712.2000.tb02090.x.

[4] Miniati M, Monti S, Pratali L, Di Ricco G, Marini C, Formichi B, et al. Value of transthoracic echocardiography in the diagnosis of pulmonary embolism: results of a prospective study in unselected patients. Am J Med 2001;110:528–35. doi:10.1016/S0002-9343(01)00693-3.

[5] Bova C, Greco F, Misuraca G, Serafini O, Crocco F, Greco A, et al. Diagnostic utility of echocardiography in patients with suspected pulmonary embolism. Am J Emerg Med 2003;21:180–3.

[6] Chen J-Y, Chao T-H, Guo Y-L, Hsu C-H, Huang Y-Y, Chen J-H, et al. A Simplified Clinical Model to Predict Pulmonary Embolism in Patients With Acute Dyspnea. Int Heart J 2006;47:259–71. doi:10.1536/ihj.47.259.

[7] Lodato JA, Ward RP, Lang RM. Echocardiographic Predictors of Pulmonary Embolism in Patients Referred for Helical CT. Echocardiography 2008;25:584–90. doi:10.1111/j.1540-8175.2008.00665.x.

[8] Kalkan AK, Ozturk D, Erturk M, Kalkan ME, Cakmak HA, Oner E, et al. The diagnostic

value of serum copeptin levels in an acute pulmonary embolism. Cardiol J 2016;23:42–50. doi:10.5603/CJ.a2015.0077.

[9] Daley J, Grotberg J, Pare J, Medoro A, Liu R, Hall MK, et al. Emergency physician performed tricuspid annular plane systolic excursion in the evaluation of suspected pulmonary embolism. Am J Emerg Med 2017;35:106–11. doi:10.1016/j.ajem.2016.10.018.

[10] Dwyer KH, Rempell JS, Stone MB. Diagnosing centrally located pulmonary embolisms in the emergency department using point-of-care ultrasound. Am J Emerg Med 2018;36:1145–50. doi:10.1016/j.ajem.2017.11.033.
